# From Rhabdomyolysis to a Lymphoproliferative Disorder: A Long Diagnostic Work-Up

**DOI:** 10.7759/cureus.99844

**Published:** 2025-12-22

**Authors:** Núria Condé Pinto, Ana Pessoa, Helga Martins, Mário Esteves

**Affiliations:** 1 Internal Medicine, Unidade Local de Saúde do Médio Ave, Vila Nova de Famalicão, PRT

**Keywords:** covid-19, dermatomyositis, immunization, lymphoma, paraneoplastic syndrome, rash, rhabdomyolysis

## Abstract

Rhabdomyolysis is characterized by myocyte necrosis with destruction of the skeletal muscle that can lead to severe complications if left untreated. Due to its multiple possible etiologies, identifying the underlying cause can be challenging, especially when the clinical presentation is atypical or evolves. We present the case of a 73-year-old man who developed progressive proximal muscle weakness shortly after COVID-19 vaccination. Laboratory tests were consistent with rhabdomyolysis. Despite supportive treatment and clinical response to corticosteroids, the underlying cause remained initially unclear. Months later, the patient developed dermatomyositis, and further investigation revealed a lymphoma, suggesting a paraneoplastic process. In patients presenting with a first episode of unexplained rhabdomyolysis, particularly when symptoms are progressive, clinicians should maintain a high index of suspicion for inflammatory myopathies. This case highlights the complexity of the diagnostic work-up for paraneoplastic dermatomyositis, especially when the initial presentation is incomplete, and underscores the importance of long-term follow-up and continued investigation to establish a definitive diagnosis and exclude neoplasms that may only become apparent years later.

## Introduction

Muscle pain and weakness are frequent reasons for admission to emergency and primary healthcare services. The differential diagnosis ranges from benign and transient conditions to more complex or severe pathologies [1]. Therefore, a thorough medical history and physical examination are crucial for the diagnostic workup and management.

Rhabdomyolysis is characterized by myocyte necrosis, leading to disruption of the sarcolemma and destruction of striated muscle with subsequent release of intracellular components into the bloodstream. It is diagnosed when creatine kinase (CK) levels exceed 1000 U/L or are more than five times the upper limit of normal. Acute kidney injury (AKI) and myoglobinuria may occur. The clinical presentation of rhabdomyolysis is variable and may be asymptomatic; however, the most common manifestations include muscle weakness, pain, and myoglobinuria. If untreated, rhabdomyolysis can lead to severe complications, including electrolyte imbalances and AKI, which may be fatal [2,3].

Causes of rhabdomyolysis are broadly categorized into genetic and acquired. Genetic causes predominantly include metabolic and mitochondrial disorders. Acquired causes are either traumatic or non-traumatic (exertional and non-exertional) [2]. It is also critical to rule out inflammatory myopathies, which can present with myalgia, muscle weakness, and elevated muscle enzymes, and may have a more insidious onset [4].

In this report, we describe a patient who initially presented with rhabdomyolysis, and whose etiology remained elusive for more than a year of follow-up. We aim to focus on the diagnostic challenge inherent to rhabdomyolysis, particularly when it represents the first nonspecific manifestation of inflammatory myopathies, and its association with neoplasms, which may present late, thereby reinforcing the importance of rigorous follow-up.

## Case presentation

A 73-year-old male, previously independent in activities of daily living, presented to the emergency department (ED) with complaints of myalgia and proximal muscle weakness. The patient's medical history included hypertension, dyslipidemia, mild aortic valve stenosis, prostate acinar adenocarcinoma classified as Gleason 6 with no current evidence of active disease, previous smoking history of 5 pack-years, and alcohol consumption of 12 grams per day. His regular medications included ramipril/hydrochlorothiazide 2.5/12.5 mg per day, and he had been on statin therapy for dyslipidemia for five years, which he discontinued two weeks before admission. Additionally, there was a long history of pigeon breeding. There were no known allergies.

The patient reported that four days after receiving the second dose of the anti-SARS-CoV-2 vaccine, he began experiencing myalgia at the inoculation site (left arm). Over the following days, myalgia extended to his right arm and thighs, accompanied by progressive muscle weakness, severely limiting arm elevation, rise from bed, and walking. He did not monitor body temperature. Three days later, he independently discontinued statin therapy. Despite this, his symptoms persisted, prompting two visits to the ED within the same week, where he was treated with anti-inflammatory agents and muscle relaxants. On the 12th day of symptoms, he returned to the ED and was diagnosed with pneumonia, based on mild infiltration observed in the lower right lung lobe on a chest X-ray and an elevated C-reactive protein (CRP) level of 12 mg/dL. He was discharged with prescriptions for amoxicillin/clavulanate and azithromycin. Five days later, he returned to the ER due to persistent generalized myalgia and worsening proximal muscle weakness.

The patient denied any similar previous episodes, including during childhood, and there was no family history of muscular disorders. He also reported no exposure to ill individuals or any epidemiological context suggestive of infection. He denied the use of any medications other than those listed, as well as herbal supplements. Additionally, he reported no recent changes in physical activity, trauma, or surgical procedures.

In the ED, the patient was alert, cooperative, and oriented. His vital signs included a blood pressure of 149/50 mmHg, mild tachycardia at 107 beats per minute, an oxygen saturation of 98% on room air, and no fever. Cardiac auscultation revealed a regular rhythm with normal heart sounds. Pulmonary auscultation was unremarkable, demonstrating a vesicular breath sound pattern without any adventitious sounds. The abdominal examination was soft, non-tender, and tympanic, with no palpable masses or signs of peritoneal irritation. Musculoskeletal examination revealed an inability to perform passive shoulder abduction or hip flexion and mild muscle swelling, particularly in the arms. Further diagnostic testing was performed, and the results are summarized in Table 1, suggesting rhabdomyolysis without signs of renal impairment.

**Table 1:** Initial laboratory tests CRP: C-reactive protein; ALT: alanine aminotransferase; AST: aspartate aminotransferase; LDH: lactate dehydrogenase; ALP: alkaline phospatase; GGT: gamma-glutamyl transferase; HPF: high power field; CK: creatinine kinase; PaO2: partial pressure of oxygen; PaCO2: partial pressure of carbon dioxide; TSH: thyroid-stimulating hormone; T4: thyroxine

Test	Patient Value	Reference Range
Blood		
Haemoglobin ( g/dL)	15.00	13.0 - 18.0
Leukocytes (uL)	29.62x10^3^	4.0 - 11.0
Neutrophils (%)	86.20	40-74
Platelets (uL)	589x10^3^	130 - 450
Creatinine (mg/dL)	0.62	0.72 - 1.25
Urea (mg/dL)	69	17.9 - 55.0
Sodium ( mEq/L)	128	136 - 145
Potassium ( mEq/L)	4.9	3.5 - 5.1
CRP (mg/dL)	9.35	0 - 0.5
Ionized calcium (mmol/L)	1.03	1.13 - 1.32
Phosphorus (mg/dL)	4.82	2.3 - 4.7
Glucose (mg/dL)	82	82 - 115
Myoglobin (ng/mL)	5588	17.4 - 105.7
CK (U/L)	2440	30 - 200
ALT (U/L)	960	5 - 34
AST (U/L)	485	0 - 55
LDH (U/L)	1021	125 - 220
ALP (U/L)	167	40 - 150
GGT ( U/L)	110	12 - 64
TSH (ng/dL)	1.09	0.35 - 4.94
T4 (µUI/mL)	1.04	0.70 - 1.48
Fibrinogen (ng/mL)	475	180 - 350
D-Dimers (ng/mL)	2582	0 - 560
Lactate (mmol/L)	1.9	<1
pH	7.5	7.35-7.45
Pa02 (mmHg)		
PaC02 (mmHg)	31	35-45
Bicarbonate (mEq/L)	24.2	22-26
Urine		
Nitrites	0	0
Proteins (mg/dL)	10	<10
Glucose (mg/dL)	0	<50
Leucocytes (HPF)	0-2	<25
Erythrocytes (HPF)	0-2	<2-5
Epithelial cells (HPF)	0-2	<0-2

A thoraco-abdominopelvic CT scan revealed diffuse bilateral pulmonary emphysema, predominantly centrilobular in the upper lobes, and mild hepatic steatosis, with no additional abnormalities. The patient was started on intravenous fluid therapy and admitted for further evaluation and to investigate the etiology of rhabdomyolysis.

The case was discussed with the Neurology team. Given the possibility of an inflammatory myopathy, a muscle biopsy was planned, and corticosteroid therapy with prednisolone at a dose of 1 mg/kg/day (70 mg/day) was initiated. Further investigations showed negative viral serologies (Table 2).

**Table 2:** Diagnosis work-up for viruses

Virus	Patient Result	Comment
Anti-SARS-CoV2 spike antibody	Positive	Previous vaccination
Anti-SARS-CoV2 IgM antibody	Negative	
Influenza A and B virus antigens	Negative	Non-infected
Human immunodeficiency virus 1 and 2 antibodies	Negative	Non-infected
Cytomegalovirus IgG antibody	Positive	Past infection
Cytomegalovirus IgM antibody	Negative	
Herpes simplex 1 IgG antibody	Positive	Previous contact
Herpes simplex 2 IgG antibody	Negative	No contact
Epstein-Barr virus IgG antibody	Positive	Past infection

Other findings included normal prostate-specific antigen (PSA), negative fecal occult blood test, and a normal thoraco-abdominopelvic CT. A high-resolution thoracic CT scan revealed bilateral centrilobular and paraseptal emphysema, predominantly affecting the upper lobes, with no nodules, ground-glass opacities, honeycombing, or infiltrative changes noted. Electromyography (EMG) indicated acute myopathy with normal nerve conduction studies. Preliminary findings from the muscle biopsy demonstrated neurogenic changes without evidence of inflammation.

During hospitalization, the patient continued on intravenous fluid therapy, diuretics, and corticosteroids. He showed both clinical and biochemical improvement, with no evidence of renal impairment. Antinuclear antibodies (ANA) were positive (titer of 1:320) with a nucleolar speckled pattern; however, the remainder of the autoimmune workup was still in progress, as were the arm MRI and muscle biopsy results. He began physical therapy and was discharged after a 20-day hospital stay, with elbow elevation limitation but no pain or signs of inflammation. He was scheduled for follow-up with internal medicine, neurology, and physical rehabilitation, with further results pending.

At the first follow-up appointment, 10 days post discharge, the patient reported continued improvement, although he remained unable to lift his arms beyond 90 degrees. He was actively undergoing physical rehabilitation and remained on 70 mg of prednisolone daily without the emergence of new symptoms. At this time, the remaining autoimmune studies returned negative results (Tables 3, 4).

**Table 3:** Anti-nuclear antibodies (ANA) panel results

Parameters	Patient Result	Comment
ANA	1:320	Positive
Pattern	Speckled and nucleolar	Negative
Mitosis	-	Negative
Quantitative detection of antinuclear antibodies (anti-dsDNA, anti-Ro-60, anti-Ro52, anti-SSB, anti-Scl70, anti-centromer, anti-Th/To, anti- Polimerase III, anti-SM, anti-RNP, anti-Ku, anti-Scl-70, anti-PM Scl 100, anti-PCNA,anti-nucleosome, anti-histones, anti-P protein, anti-mithocondria, anti-DFS 70)	<20 UQ	Negative

**Table 4:** Myositis-specific antibodies panel results

Myositis specific antibodies	Result
Anti-Mi-2	Negative
Anti-Mi-2 BETA	Negative
Anti-TIF1 GAMA	Negative
Anti-MDA 5	Negative
Anti-NXP 2	Negative
Anti-SAE 1	Negative
Anti-Ku	Negative
Anti-PM Scl 100	Negative
Anti-PM-Scl 75	Negative
Anti-Jo-1	Negative
Anti-SRP	Negative
Anti-PL-7	Negative
Anti-PL-12	Negative
Anti-EJ	Negative
Anti-OJ	Negative
Anti-Ro - 52	Negative

**Figure 1:**
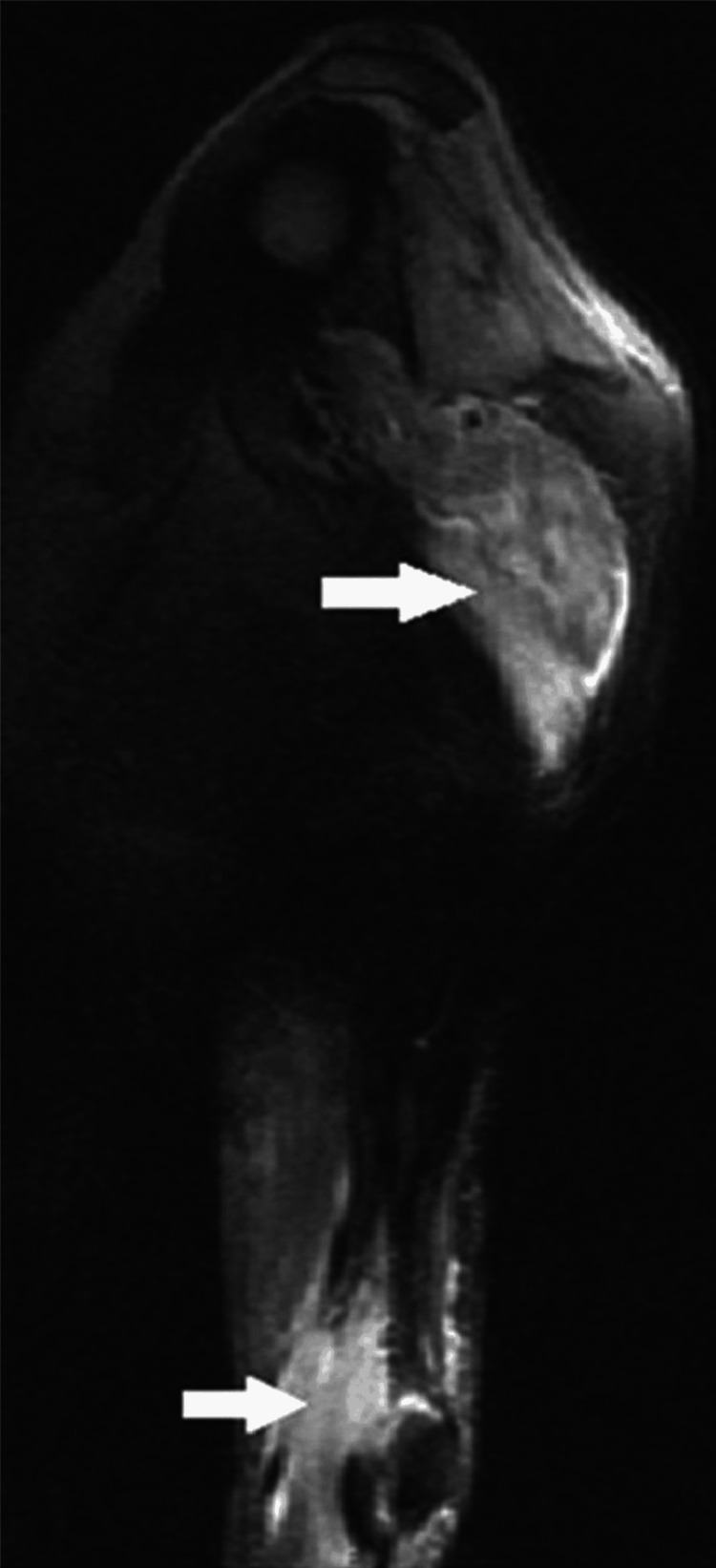
Muscle MRI (T2-STIR) showing inflammatory changes across nearly all muscle groups of left arm and shoulder (edematous hyperintensity and dissociation of muscle fibers STIR: short tau inversion recovery

MRI findings indicated severe inflammatory changes across nearly all muscle groups in the left arm and shoulder, characterized by prominent edematous hyperintensity and dissociation of muscle fibers. There were no signs of muscle liquefaction, mass lesions, adenopathy, or osteoarticular changes (Figure 1). The muscle biopsy demonstrated mild and non-specific alterations, with no definitive inflammatory changes. The anti-Chlamydia psittaci IgM antibody was negative.

Two weeks later, he developed new-onset dysphagia for solid foods, prompting an otolaryngology evaluation. This symptom was attributed to myositis, and speech therapy was initiated, resulting in symptomatic improvement. Subsequent assessments demonstrated improvement in cytolysis, with complete normalization after one month, permitting a reduction in prednisolone dosage to 10 mg daily. A follow-up EMG showed no abnormalities indicative of peripheral nerve or persistent muscle lesions, but could not completely rule out resolving myopathic changes. An echocardiogram revealed mild atrial dilation without other pathological findings. Comprehensive endoscopic evaluation of the upper and lower gastrointestinal tracts ruled out malignancy.

**Figure 2:**
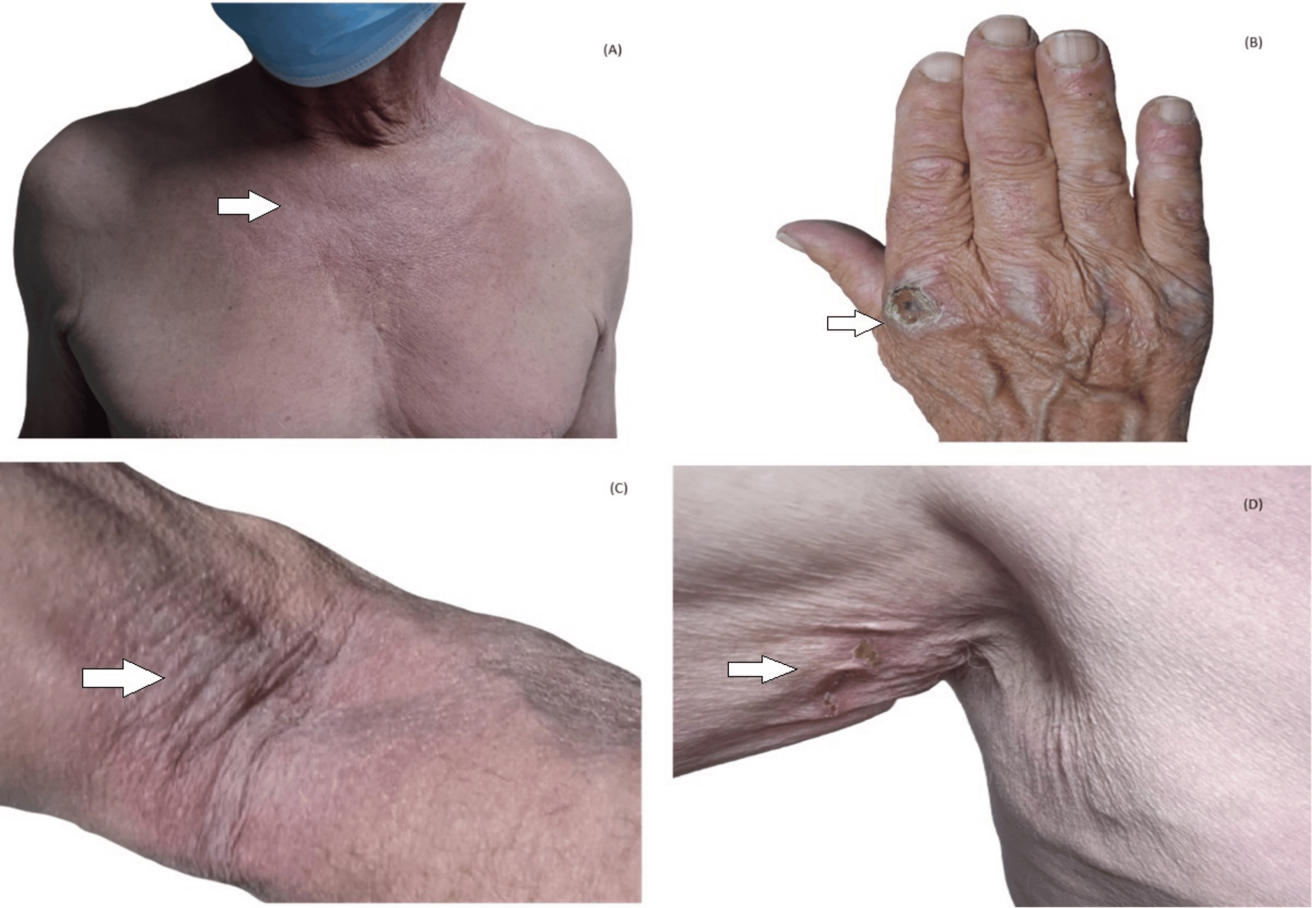
Skin manifestations: Dermatomyositis typical "V" shape violaceous neck and chest erythema (A), Gottron papules (B) and flexure rash (C,D)

The patient continued to improve, allowing for extended intervals between follow-up appointments. However, after 10 months, he reported new symptoms including a rash on the neck, hands, flexural areas of the arms, and axillae, along with nodular lesions on the extensor surfaces of the fingers (Figure 2).

He also experienced worsening limb weakness without associated myalgia and an increase in dysphagia. Following a recent neurology consultation, his prednisolone dosage was increased to 40 mg daily. Laboratory tests revealed positive ANA with a speckled and nucleolar pattern, but no evidence of muscle cytolysis. Given these findings, amyopathic dermatomyositis was considered.

The patient was referred to dermatology, where a skin biopsy was performed, revealing non-specific morphological changes. Hydroxychloroquine was initiated, and the patient was subsequently referred to rheumatology, where treatment with methotrexate and nifedipine was started.

A PET scan did not reveal any suspicious neoplastic foci, and a repeat thoraco-abdominopelvic CT was unremarkable. Serial laboratory tests showed stable blood counts, normal cytolysis markers, and an unchanged protein profile, with persistent negative results for most autoimmune markers except for ANA at a titer of 1:320 with a nuclear and speckled pattern.

**Figure 3:**
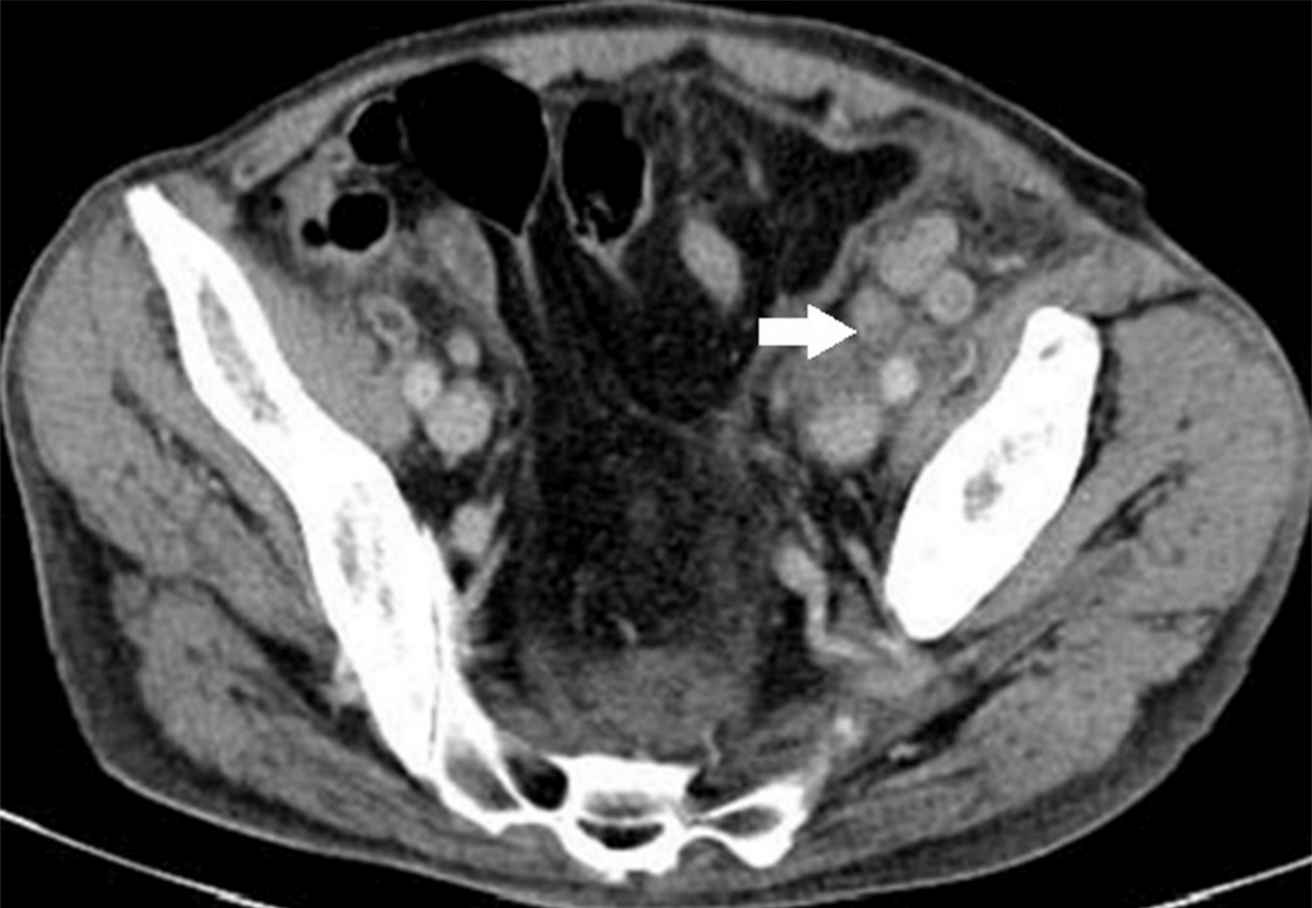
Abdominal CT showing multiple adenopathies, specifically iliac lymphadenopathies, the largest measuring 2 cm in short axis.

Five months later, the patient presented with worsening cutaneous rash, now including erythroderma. He also reported asthenia and abdominal and lower back pain. An abdominal ultrasound was planned but not performed due to his clinical deterioration. He sought emergency care, and an abdominal CT scan revealed multiple para-aortic and iliac lymphadenopathies and free fluid (Figure 3). Further investigation confirmed the diagnosis of anaplastic lymphoma, and the patient passed away a few months later. 

## Discussion

This case underscores the complexities and challenges inherent to the diagnosis workup of a patient presenting with rhabdomyolysis, particularly in cases of inflammatory muscle diseases, which can have highly variable and sometimes indolent presentations. The initial symptoms of muscle weakness and rhabdomyolysis led to an exhaustive diagnostic workup, which ultimately clarified the diagnosis as dermatomyositis. While cutaneous manifestations were absent initially, they do not exclude the diagnosis, as they may appear later [5].

In this patient, traumatic and non-traumatic exertional causes were excluded based on history and physical examination. Genetic causes were deemed unlikely given the late onset of symptoms and the absence of a family history. Immune-mediated necrotizing myopathy, potentially related to statin use, was considered less likely due to the long duration of statin therapy and biopsy results. No infectious agents were identified. The muscle biopsy results and negative autoimmune studies (although collected before corticosteroid introduction) were limiting factors in establishing an initial diagnosis of inflammatory myositis. Despite numerous antibodies associated with dermatomyositis, some cases remain seronegative. Muscle biopsy remains crucial for distinguishing between different types of myositis, with antibodies playing a role in diagnosis, subclassification, and understanding the clinical course and malignancy risk. However, the sensitivity of the biopsy could be limited due to the technique and quality of the sample, which could explain the discrepancy between it and the MRI findings of important inflammation [6,7]. The initiation of corticosteroid therapy before more conclusive results could be questioned; however, this decision was made based on the clinical presentation and significant symptomatology, as well as our clinical setting, where results can be delayed.

Regarding neoplasia, dermatomyositis is frequently diagnosed in relation to cancer, either before, concurrently with, or after cancer detection, with literature suggesting a rate of association between the two entities of approximately 30%. Regular surveillance is advised for patients with dermatomyositis, especially when initial screening is unremarkable, as malignancy most often manifests within the first three years but can occasionally present later. However, consensus on the strategy for cancer screening after a benign initial study is still lacking [8,9]. 

Finally, we question the temporal association between vaccination and the patient’s clinical course. While the immunogenic potential of vaccines as triggers or exacerbators of inflammatory diseases has been more extensively studied for other vaccines, reports of anti-SARS-CoV-2 vaccination preceding rhabdomyolysis or myositis are limited, and mostly case reports [10-12]. Considering this patient’s advanced age, history of prostate cancer, and the later diagnosis of lymphoma, the findings are more consistent with paraneoplastic myositis.

## Conclusions

The diagnostic workup of rhabdomyolysis may be challenging due to its broad range of potential etiologies. Dermatomyositis is a rare, heterogeneous condition with a highly variable clinical course. Some factors, such as drugs, infections, and vaccinations, should be taken into consideration when searching for a potential trigger. Particularly in elderly patients, it is often associated with underlying malignancies. The diagnosis can be challenging due to its variable presentation, the limitations of diagnostic tests such as muscle biopsy and autoantibody panels, and the sometimes indolent or delayed presentation of neoplasia. An integrated approach combining clinical evaluation with periodic reassessment, laboratory tests, and imaging is crucial for accurate diagnosis. Those patients require vigilant follow-up, particularly when initial cancer screenings are negative, to provide a timely diagnosis and intervention.
